# T908 Polymeric Micelles Improved the Uptake of Sgc8-c Aptamer Probe in Tumor-Bearing Mice: A Co-Association Study between the Probe and Preformed Nanostructures

**DOI:** 10.3390/ph15010015

**Published:** 2021-12-23

**Authors:** Romina Castelli, Manuel Ibarra, Ricardo Faccio, Iris Miraballes, Marcelo Fernández, Albertina Moglioni, Pablo Cabral, Hugo Cerecetto, Romina J. Glisoni, Victoria Calzada

**Affiliations:** 1 Área de Radiofarmacia, Centro de Investigaciones Nucleares, Facultad de Ciencias, Universidad de la República, Montevideo 11400, Uruguay; romi.castelli@gmail.com (R.C.); pabloc7@gmail.com (P.C.); 2 Department of Pharmaceutical Sciences, Faculty of Chemistry, Universidad de la República, Montevideo 11800, Uruguay; mi.cebiobe@gmail.com; 3 Área Física, DETEMA, Facultad de Química, Universidad de la República, Montevideo 11800, Uruguay; rfaccio@fq.edu.uy; 4 Inmunología Clínica, (BIOCLIN) y Biotecnología, Polo Tecnológico de Pando-Facultad de Química, Universidad de la República, Montevideo 11800, Uruguay; iris.miraballes@gmail.com; 5 Laboratorio de Experimentación Animal, Centro de Investigaciones Nucleares, Facultad de Ciencias, Universidad de la República, Montevideo 11400, Uruguay; xxmferna@gmail.com; 6 Instituto IQUIMEFA UBA-CONICET, Departamento de Farmacología, Facultad de Farmacia y Bioquímica, Universidad de Buenos Aires, CABA, Buenos Aires C1113AAD, Argentina; bmoglio2015@gmail.com; 7 Instituto NANOBIOTEC UBA-CONICET, Departamento de Tecnología Farmacéutica, Facultad de Farmacia y Bioquímica, Universidad de Buenos Aires, CABA, Buenos Aires C1113AAD, Argentina

**Keywords:** Sgc8-c aptamer, probe, polymeric micelles, liposomes, active targeting

## Abstract

Aptamers are oligonucleotides that have the characteristic of recognizing a target with high affinity and specificity. Based on our previous studies, the aptamer probe Sgc8-c-Alexa647 is a promising tool for molecular imaging of PTK7, which is an interesting biomarker in cancer. In order to improve the delivery of this probe as well as create a novel drug delivery nanosystem targeted to the PTK7 receptor, we evaluate the co-association between the probe and preformed nanostructures. In this work, preformed pegylated liposomes (PPL) and linear and branched pristine polymeric micelles (PMs), based on PEO–PPO–PEO triblock copolymers were used: poloxamer F127^®^ and poloxamines T1307^®^ and T908^®^. For it, Sgc8-c-Alexa647 and its co-association with the different nanostructures was exhaustively analyzed. DLS analysis showed nanometric sizes, and TEM and AFM showed notable differences between free- and co-associated probe. Likewise, all nanosystems were evaluated on A20 lymphoma cell line overexpressing PTK7, and the confocal microscopy images showed distinctness in cellular uptake. Finally, the biodistribution in BALB/c mice bearing lymphoma-tumor and pharmacokinetic study revealed an encouraging profile for T908-probe. All data obtained from this work suggested that PMs and, more specifically T908 ones, are good candidates to improve the pharmacokinetics and the tumor uptake of aptamer-based probes.

## 1. Introduction

Aptamers are small, single-strain oligonucleotides that can bind a wide range of ligands with high affinity and specificity [1]. Typically, the dissociation constant (Kd) for aptamer–target complexes is in the high pico-molar to low nano-molar range [2,3]. Aptamers can be selected against proteins, peptides, dyes, metal ions, viruses, bacteria, toxins, and whole cells, even against non-immunogenic molecules. Functionally, aptamers are similar to antibodies; however, aptamers have an ease of synthesis and easy chemical modification that allows conjugation with a variety of molecules and stability at a higher temperature. The synthetic manufacturing of aptamers allows minimal batch-to-batch variation [4]. Their physicochemical characteristics and small sizes allow the precise recognition of cellular elements and high tissue penetration [4,5]. In addition, aptamers are non-immunogenic and non-toxic [6]. Thus, aptamers are emerging as powerful tools for the development of biopharmaceutics. Currently, many aptamers are in different stages of clinical trials [7]. Sgc8-c, a 41-nucleotide DNA aptamer that recognizes specifically the PTK7 receptor was described by Shangguan et al. [8,9]. PTK7 overexpression has been reported in colon cancer [10,11], breast cancer [12], lung cancer [13], acute myeloid leukemia, acute lymphoid leukemia [14,15], melanoma [14,15], ovarian carcinoma, prostate cancer, lymphoma [14], glioma, and liposarcoma [16], among others. The association of aptamers with nanostructures offers huge opportunities in the research fields of diagnostics and therapeutics [17,18]. In the last decades, biocompatible nanostructures have been developed, due their particular properties as size, stability, large surface, and highly reactivity that makes them interesting for the biomedicine field. Indeed, nanomedicine has been able to overcome some of the principal limitations emerged with the use of therapeutic and diagnostic agents in the last few years [17,19,20,21,22,23,24,25,26].

Liposomes are one of the most extensively used nanostructures due their lipid-based spherical-shaped unique vesicular structure with sizes between 50 and 1000 nm [19,20,21,22]. These vesicles are composed of a lipid bilayer that forms a hollow sphere encompassing an aqueous phase. Thereby, different bioactive molecules can be encapsulated within liposomes in either the aqueous compartment (hydrophilic ones) or within the lipid bilayer (hydrophobic ones) [20,21,22,23,24]. They are used to protect different active molecules such as drugs, DNA and RNA molecules, plasmids, and proteins from degradation in vivo; control the substance release, biodistribution modification, and target drug delivery to the site of disease; and enhance its solubility and bioavailability [23,24,25,26]. On the other hand, polymeric micelles (PMs) represent one of the most versatile nanotechnology platforms in the last few decades [27,28,29,30,31,32]. PMs are formed by the self-assembly of copolymeric amphiphiles having two primary domains, a hydrophobic core and a hydrophilic shell [27,28,29,30,31,32]. Due to the great flexibility of tailoring their molecular weight, hydrophilic–lipophilic balance (HLB), small size, architecture, surface chemistry, and shape, they have great potential as a target delivery nanosystem [27,28,29,30,31,32]. Poloxamers and poloxamines are the most investigated variant of nonionic polymers for the development of drug delivery and vaccine adjuvants. They are formed by a linear triblock of poly(ethylene oxide)-poly(propylene oxide) (PEO-PPO) and are found as unimers in the aqueous medium below the Critical Micelle Concentration (CMC) [31,32,33,34,35]. Above the CMC, copolymer aggregate to form nanomicelles consisting of a hydrophobic core of PPO chains, while the PEO chains form the hydrophilic shell [31,32,33,34,35]. At certain concentrations and temperatures, some poloxamers of high molecular weight are able to organize themselves, forming gel-like structures and allowing a sustained release of drugs and proteins [36,37,38]. The selection of the poloxamer type depends on the properties of the antigen, finding a greater interaction with hydrophilic soluble proteins when copolymers have a higher percentage of PEO, while hydrophobic proteins with transmembrane regions interact better with copolymers with a higher percentage of PPO [38,39,40,41,42,43].

The adsorption of serum proteins to the nanostructure surface plays a critical role in the clearance of these from the blood circulation [20,21,22]. Owing to this, the surface modification with poly(ethylene glycol) (PEG) is well known not only for maintain the particle structure, but also, it could be useful, for example, to sustain oligonucleotide–ligands even in a serum environment [21]. Pegylation makes nanostructures less sensitive to opsonization and prolongs their half-life within the organism [20,21,22]. 

Based on our previous studies, an aptamer probe Sgc8-c-Alexa647 (Figure S1) is a promising tool for the molecular imaging of PTK7, which is an interesting biomarker in cancer [14,44,45,46,47]. Thus, with the aim to improve the delivery of the probe as well as explore additional drug delivery strategies, we evaluate the co-association between the probe and nanostructures. Since it is crucial to functionalize aptamers without affecting their ability to fold into this binding-competent structure [48], here, we performed the co-association using preformed pegylated liposomes (PPL) and pristine poloxamer-based (F127^®^) and pristine poloxamine-based (T908^®^ y T1307^®^) PMs. The probe was evaluated in both type of nanostructures, and the physicochemical characteristics of the products were determined. Finally, as in vivo proof of concept, pharmacokinetic and biodistribution studies were carried out.

## 2. Materials and Methods

### 2.1. Materials

The desalted 5′-(6-aminohexyl)-modified Sgc8-c aptamer (*M_w_* = 12.8 kDa, 5′-6-aminohexyl-ATC TAA CTG CTG CGC CGC CGG GAA AAT ACT GTA CGG TTA GA -3′, Sgc8-c-NH_2_) was purchased from IDT Technologies (Integrated DNA Technologies Inc., Coralville, IA, USA). The probe Sgc8-c-Alexa647 (*M_w_* = 13.7 kDa, Figure S1A) was prepared and purified using Sgc8-c-NH_2_ and an activated-Alexa647 (Thermo Fisher Scientific Inc., Waltham, MA, USA), as previously it was reported by Calzada et al. [14,45]. Finally, the probe was lyophilized without any additives and stored at −20 °C. Preformed pegylated liposomes (PPL) were gently gifted by MR-Pharma S.A. (Buenos Aires, Argentina). Micelle-forming copolymers—(i) poloxamer-based Pluronic^®^ 127 (F127, *M_w_* = 12.6 kDa, PEO 70% *w*/*w*, HLB 18), (ii) pH-dependent poloxamine-based Tetronic^®^ 1307 (T1307, *M_w_* = 18 kDa, PEO 70% *w*/*w*, HLB 27) and (iii) 908 (T908, *M_w_* = 25 kDa, 80% *w*/*w* PEO, HLB 31)—were provided by BASF Corporation (New Milford, CT, USA, Figure S2A,B).

### 2.2. Co-Associations of the Probe with Preformed Nanostructures

Unilamellar bilayer PPL, composed by cholesterol (2 mg/mL), hydrogenated soy phosphatidylcholine (6 mg/mL), and phosphatidylethanolamine (4 mg/mL) grafted to a short chain of polyethylene glycol (*M_w_* ~2 kDa), were gently gifted and manufactured using two cycles by a high-pressure homogenizer (PandaPlus1000, Gea Lab) by MR-Pharma S.A. (Buenos Aires, Argentina).

Free-PMs were prepared according to the method described by Glisoni et al. [32,33,34,35]. Briefly, the copolymers were hydrated overnight at 4 °C to 70% of the final volume using Milli-Q water (pH 6.0, for F127) or PBS (pH 7.4, for T1307 and T908), and after that, the volume was completed, at room temperature, in order to obtain aqueous dispersions of PMs at 10% *w*/*v*. Then, the co-association of Sgc8c-Alexa647 (probe) to preformed nanostructures was carried out by incubating it, previously lyophilized (50 μg), to one milliliter of (i) preformed pegylated liposomes (PPL) and (ii) pristine polymeric micelles (PMs, 10% *w*/*v*) based on poloxamer (F127) and poloxamines (T1307 and T908) for 30 min at 4 rpm in the dark and at room temperature. In addition, a vesicular exclusion column (VEC) qEVoriginal, 70 nm (ICO-70, IZON), was used to purified the co-associated to the non-associated probe between probe and PPL. To elute, Milli-Q water was used. Finally, the eluted fractions (0.5 mL) were measured in a Varioskan Flash, fluorescence intensity (IF) λ_Em/Ex_ 675/640), and absorbance (A), λ = 650 nm in order to evaluate the final concentration associated to PPL. PMs–probe nanosystems did not require any type of additional purification. Finally, the nanosystems/probe obtained in each case were named as (i) PPL-probe, (ii) F127-probe, (iii) T1307-probe, and (iv) T908-probe, respectively.

### 2.3. Nanosystems Characterization

#### 2.3.1. Particle Size and Zeta Potential Measurements

The hydrodynamic diameter (D_h_), the polydispersity index (PDI), the zeta potential (Z-Potential), and the stability over time (stored at 4 °C in the dark for 24 h) of the nanosystems/probe at 25 and 37 °C, were measured by Dynamic Light Scattering (DLS) using a Nano-ZS Zetasizer with Non-Invasive Back Scatter (NIBS^®^) technology (Malvern Ltd., Malvern, UK). As controls, free-probe, free-PPL, and free-PMs were analyzed in the same conditions. The determinations were performed at a fixed scattering angle of 173° and fixed laser position 4.65 mm, He-Ne laser (633 nm), and a digital correlator (ZEN3600). For Z-Potential, laser Doppler micro-electrophoresis is used. The refraction indexes (IR) were 1.48 (PPL) and 1.33 (PMs), and viscosities were between 0.8869 and 0.8876 cP at 25 °C and between 0.6850 and 0.6875 cP at 37 °C. Each sample was analyzed in triplicate, and the values were counted as an average with six measurements each.

#### 2.3.2. Transmission Electron Microscopy (TEM)

For TEM observations, a ZEISS EM109 TEM (Oberkochen, Germany) was used. Free- and co-associated probe nanosystems (10 µL) were placed into a carbon grid coated with a hydrophilic acrylic resin of low viscosity (LR-White) during 5 min. After the incubation, the excess of sample was removed with tissue paper and dried in the air for 10 min before placed into the microscope. Then, the grid was coated with uranyl acetate (50 µL, 2% *w*/*v* deionizing water) during 180 s. Finally, the grid was dried in the air at room temperature for 15 min. Images were taken at 80 kV, at room temperature, and using low-dose imaging conditions with a CCD digital camera ES1000W Erlangshen^TM^ high speed and 11 MP (Model 785, Gatan GmbH, München, Germany). The diameter and size distribution of the nanosystems were estimated using the TEM AutoTune™ software (Gatan Digital Micrograph^®^ software, Gatan GmbH).

#### 2.3.3. Atomic Force Microscopy (AFM)

Free-PPL and PPL-probe were used as we prepared. For the free-PMs and PMs-probe, a 1:300 dilution using MilliQ-water was needed. First, 2–5 μL of each sample were deposited onto a mica surface (PPLs) and silicon surface (PMs). Then, they were dried under nitrogen gas. Measures were performed with a Confocal Raman Microscopy plus AFM (Alpha 300-R A, WITec, Ulm, Germany) operating in a non-contact mode by tapping using a reflective coated silicon cantilever with an elasticity constant of 42 N/m with a resonance frequency of 285 Hz. The images were processed with the Project FOUR 4.1 WITec software. 

### 2.4. In Vitro Release of the Probe from Nanosystems

The in vitro release of Sgc8c-Alexa647 (probe) was studied mainly to ensure the stability of the co-association between probe, PPL-probe, and PMs-probe. The study was done using regenerated cellulose dialysis membranes (MWCO of 15,000 g/mol, Spectra/Por^®^, Spectrum Laboratories, Inc., Rancho Dominguez, CA, USA) and constant agitation at 25 °C, as we previously assayed for similar nanosystems [49,50,51]. Briefly, 2 mL of PPL-probe and T908-probe (T908 *M_w_* = 25 kDa) were loaded inside each dialysis bag and placed into the adequate release medium (8 mL) at room temperature in the dark at 100 rpm: (i) Milli-Q water at pH 6 for PPL-probe and (ii) PBS pH 7.4 for T908-probe. Samples were drawn at preset time intervals from the receiver solution (0, 0.25, 0.5, 0.75, 1, 2, 3, 4, 24, 25, 28, 30, and 44 h of release) and equal amounts of Milli-Q water or PBS were replaced to keep release sink conditions and a constant volume, as appropriate. The amount (%) of accumulated released probe was measured by the following of the absorbance at 650 nm (UV-Vis Shimadzu 1800). Then, zero-order, first-order, Higuchi, and Korsmeyer–Peppas kinetic models were examined. The correlation coefficients (R^2^) were calculated in each case for obtaining the best fitted model to explain the release kinetics and mechanism of probe release. In addition, free-probe (*M_w_* = 13.7 kDa) was also released from the dialysis bag in both release medium as the control of the process.

### 2.5. Cellular Uptake

Free and co-associated probe nanostructures were studied by confocal microscopy (Zeiss LSM 800, software ZEN Blue 2.3, AiryScan Processing) using an endocytosis marker. The B-cell lymphoma cell line A20 (American Type Culture Collection) was growing in suspension in RPMI-STA, 10% FBS (Capricorn Scientific GmbH, Ebsdorfergrund, Germany) at 37 °C, 95% relative humidity, and 5% CO_2_. For the experiment, the culture medium was removed by centrifugation for 3 min at 800 rpm and washed twice with phosphate-buffered saline (PBS). The nanosystems were incubated with 5000 cells for 0.25, 0.5, and 1 h at a 37 °C in a final volume of 400 μL. After the incubation time, the mixture was removed, and cells were washed twice with 1 mL of ice-cold PBS. Cells were fixed in formaldehyde 4% and washed with PBS. Finally, cells were transferred to a slide and were centrifuged for 4 min at 800 rpm in cold and additionally labeled with nuclear Hoechst 33342 (1:100, ImmunoChemistry Technologies LLC, Bloomington, MN, USA) and an early endosomal marker Rab5 (1:100, C8B1 mAb 3547, Cell Signaling Technology Inc., Danvers, MA, USA), using a secondary antibody with Alexa Fluor^®^ 488 (1:500, ab150077 Abcam plc, Cambridge, UK). ProLong^TM^ Antifade mountant (Thermo Fisher Scientific Inc., Waltham, MA, USA) was applied directly to fluorescently labeled cell samples on microscope slides to protect the dyes from photobleaching during fluorescence microscopy experiments. Images were acquired with 405, 488, and 640 nm lasers.

### 2.6. Biodistribution in B-Cell Lymphoma Bearing Mice Model

BALB/c female mice (6–8 weeks of age) weighing 20–25 g were produced and provided by the Reagent Unit for Experimental Biomodels (URBE, Facultad de Medicina, Universidad de la República, Montevideo, Uruguay). The authors state that they followed the principles outlined in the Declaration of Helsinki for all animal experimental investigations. Animals were housed in wire mesh cages at 20 ± 2 °C with 12 h artificial light–dark cycles. The animals were fed ad libitum to standard pellet diet and water and were used after a minimum of 3 days of acclimation to the housing conditions. All protocols for animal experimentation were carried out in accordance with procedures authorized by the Ethical Committee for Animal Experimentation, Uruguay, by whom this project was previously approved (CEUA-FCien-UdelaR Protocol number 240011-001904-17). The biodistributions of PPL-probe and T908-probe were determined in female BALB/c mice bearing A20 B-Cell lymphoma. Previously, mice were injected subcutaneously with 5 × 10^5^ of A20 cells in the flank. Approximately 15 days after, the tumors were palpable to perform the experiment. Each animal was injected with a unique bolus dose of free-probe, PPL-probe, and T908-probe in a final volume of 200 µL (50 μg/mL of the probe in each nanosystem). Mice (n = 5) were euthanized at 0.5, 2, and 24 h post-injection, and ex vivo images with tumors and organs were acquired in the In-Vitro MS FX Pro (Bruker) equipment, X-rays, and fluorescence (λ_Ex_ 650). The data processing was performed with the Molecular Imaging Software v.7.13 (Bruker).

### 2.7. Pharmacokinetic Studies in Mice

A pharmacokinetic study of T908-probe was performed in healthy female BALB/c mice (n = 5). A unique dose, in bolus, of T908-probe (50 µg/mL of the probe) was injected in the tail vein with 200 µL of final volume. Mice were disposed into a metabolic cage, and blood samples were collected from eyeballs at 0.25, 0.5, 1, 2, 3.5, 6, 18, 24, and 48 h post-injection. Each sample of blood was mixed with EDTA.2Na (1.5 µg/µL). The samples were measured by imaging equipment (In-Vitro MF MS-XPro, Bruker) with an emission filter of 640 nm. The data analysis was processed by Bruker software and ROIs were quantified. The same procedure was performed with free-probe (50 µg/mL) (n = 5). Pharmacokinetic analysis of measured fluorescence divided by the sample volume was performed from a population approach by nonlinear mixed effects modeling (NLME) using MonolixSuite 2020R2 (Lixoft SAS, Antony, France). Model development was informed by diagnostic metrics and graphics. The corrected Bayesian Information Criterion (BICc) computed from the estimated log-likelihood was used to optimize model parsimony, assessing the tradeoff between data fit and model complexity. Basic goodness of fit plots included observations versus individual and population predictions, residuals versus time and versus the probe concentration, and the distribution of residual error. In addition, simulation-based diagnostics such as visual predictive check (VPC) and normalized prediction distribution errors (NPDE) were also considered. A covariate analysis was performed assessing the impact of the formulation on probe disposition parameters after the base model was defined. This was performed as univariate analysis on specific pharmacokinetic parameters, which is guided by the observed difference in random effects between both formulations (i.e., the discrepancies between individual parameters and the typical value). To assess the statistical significance of the covariate effect, the log-likelihood ratio was implemented: a significant improvement in data fit (*p* < 0.05) is detected by a drop in the objective function (−2*log-likelihood) of 3.84 points. In addition, a Wald test was performed to assess the significance of the estimated effect. 

### 2.8. Statistical Analysis

The statistical analysis was performed by one-way ANOVA combined with Bonferroni’s post hoc test (Bonferroni’s multiple comparison test); P values smaller than 0.05 (*p* < 0.05) were considered statistically significant. The software used was GraphPad Prism version 5.00 for Windows (GraphPad Software Inc., San Diego, CA, USA). Statistical analysis for animal experiment was performed using Student’s t test because the data are independent and have a normal distribution. 

## 3. Results 

### 3.1. The Initiative

The novel probe (Sgc8c-Alexa647, Figure S1A) with high affinity to the PTK7 receptor has the following in its structure: (i) a principal domain with hydrophobic characteristics from the fluorophore-portion Alexa647 and (ii) a purely hydrophilic domain from the main oligonucleotide structure; thus, in this work, we propose to exhaustively investigate its co-association with two structurally different types of nanostructures (PPL and PMs) at different hydrophilic and hydrophobic key points of these nanosystems (Figure S1A,B). Therefore, we hypothesized that the probe co-association with PPL and PMs would increase its in vivo mean residence time (MRT) and favor the accumulation into tumors due to the enhanced permeation and retention effect (EPR effect) and the cellular uptake by the active targeting through the PTK7 receptor overexpressed on lymphoma tumors.

### 3.2. Co-Associations of the Probe with Preformed Nanostructures

To further study the existence of probe non-co-associated to the nanosystems, we proceeded to evaluate after the appropriate incubation, the elution of PPL-probe (50 μg/mL) into a vesicular exclusion column (VEC) to identify and separate free-probe from the liposomal fraction. Two separated fractions were obtained after elution in each case. Free-probe was eluted between 7 and 9 mL volume of Milli-Q water. The liposomal fraction corresponded at 5 mL of elution volume, which is consistent with the larger particle size. After the adequate quantifications, it was estimated that 80% of the probe (40 μg/mL) co-associated to PPL post-incubation and post-purification with VEC (PPL-probe-c). Free-probe control was also evaluated through VEC, obtaining the same elution fraction. Since the self-assembly between the probe and the PMs is spontaneous above the CMC of each copolymer, the PMs-probe nanosystems did not require any type of additional purification and were used as obtained after the adequate incubation.

### 3.3. Nanosystems Characterization

Free-probe, free-PPL, free-PMs, PPL-probe, and PMs-probe were evaluated by Dynamic Light Scattering (DLS). The hydrodynamic diameter (D*_h_*), polydispersity index (PDI), and zeta potential (Z-potential) were measured in Milli-Q water and PBS, as appropriate, at 25 and 37 °C. The resulting data are showed as mean ± standard deviation (±S.D.) in Table 1 and Table 2.

DLS analysis at 25 and 37 °C showed nanometric sizes in the range of 192 and 264 nm for PPL-probe and between 11 and 32 nm for PMs-probe (Table 1 and Table 2). Free-PPL or PPL-probe showed monomodal distributions in all cases (Table 1). Z-average sizes were obtained from the cumulative analysis for free-PPL, as they were received from the high-pressure homogenizer (PPL) and those purified by VEC (PPL-c) were between 172 (PPL-c) and 190 (PPL) nm at 25 °C and between 184 (PPL-c) and 202 (PPL) nm at 37 °C, without significant differences according to both temperatures and purification processes (Table 1). A reduction in the Z-average size was observed for PPL-probe-c (Table 1). We suggest that PPL-probe-c is recovered in the collected fractions by VEC with a more limited range of Z-average sizes (between 187 and 204 nm at 25 and 37 °C, Table 1) compared to PPL-probe original sizes (between 176 and 236 nm at 25 and 37 °C, Table 1). Moreover, differences were noticeable by the CONTIN analysis, where we observed hydrodynamic diameters (D*_h_*) in the range of 249 and 257 nm at 25 and 37 °C for free-PPL, while PPL-probe (50 μg/mL of the probe) showed D*_h_* of 192 ± 19 nm and 264 ± 8 at 37 and 25 °C, respectively (Table 1). After VEC purification, free PPL-c showed sizes between 213 and 221 nm at 25 and 37 °C compared to PPL-probe-c with D*_h_* in the range of 226 and 241 nm at the same temperatures. PDI is the parameter that indicates the degree of polydispersity in the sample. All the PDI values obtained corresponded to values of less than 0.5, between 0.151 and 0.417, indicating that they were all suitable for monomodal distribution without significant changes after the increase in body temperature (Table 1). Free-probe showed Z-average sizes between 12 and 13 nm in Milli-Q water at 25 and 37 °C (Table 1). The Z-potentials of free-PPL and PPL-probe were presented in a range of −29 to −35 mV before VEC purification at 25 and 37 °C, while after VEC, they displayed values between −61 and −65 mV to the detriment of being in their free-form or being co-associated with the probe at 25 or 37 °C, showing a marked increase in stability after VEC by the rise repulsion of charges (Table 1). Z-potentials become more negative after VEC, and the stability of the colloidal dispersion dramatically increases (Table 1).

PEO–PPO copolymers, pristine free-F127, -T1307, and -T908 (Figure S2A,B) showed a typical population size < 8 nm in all cases, corresponding to the unimer form measured in Milli-Q water or PBS at 25 °C (see Table 2). Nevertheless, at 37 °C, the populations take on hydrodynamic sizes between 19 and 90 nm (Table 2 and Figure S3). Even more, after the co-associations between the probe and PMs, PMs-probe showed significant reductions in their D*_h_* at 25 and 37 °C in the range of 11 and 32 nm (Table 2 and Figure S3). The PDI values were also maintained, confirming the stabilization of the nanosystems (PDI = 0.271–0.502). The second population with D*_h_* of > 100 nm (between 166 and 171 nm; see Table 2) decreased in PBS at 37 °C. According to the D*_h_*, F127-based PMs in water at 25 °C showed two populations, one with D*_h_* of approximately 5 nm and the other of 44 nm. However, after the co-association, a unique population of 32 nm was observed without significant changes in PDI and Z-potential with a charge overlap that suggests the co-association at the surface. Similar results were observed working with T1307-probe in PBS, predominating (≈81%) a population with D*_h_* of 24 nm at 25 °C and of 26 nm (100%) at 37 °C. For the co-association T908-probe, in PBS, it observed a main population (≈94%) of 11 nm and a unique population of 20 nm at 37 °C (Table 2 and Figure S3). On the other hand, the clear presence of fluorescence observed for all PMs-probe in the average graphs of correlation coefficients in comparison with their free counterparts should be noted (Figure S3, graphs on the right).

In all cases, a charge overlap is observed in the Z-potentials after the co-associations between probe and PMs, with values from −8 to −3 mV (typical Z-potential of free-PMs) and from −4 to −2 mV (PMs-probe, Table 2).

**Figure 1:**
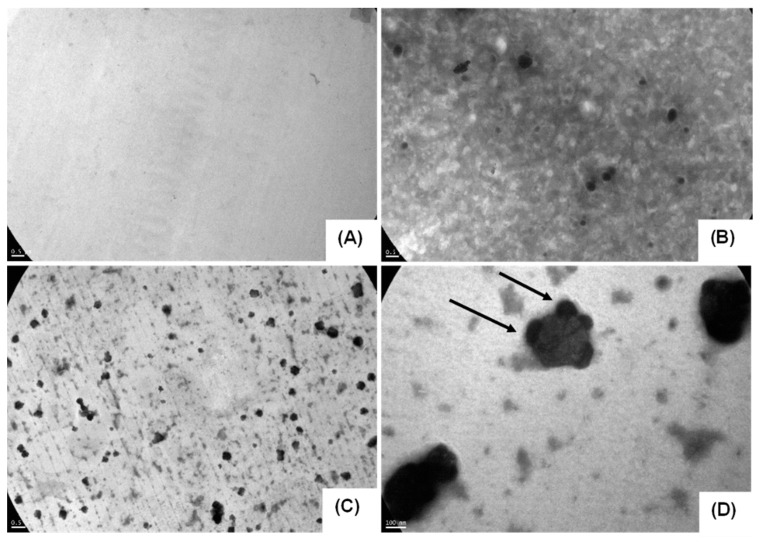
TEM images of (**A**) free-probe, (**B**) free-PPL, (**C**) PPL-probe, and (**D**) 85,000× magnification of PPL-probe. Scale bar: (**A**–**C**) 500 nm and (**D**) 100 nm. Arrows in (**D**) point out the probe on the surface of PPL.

**Figure 2:**
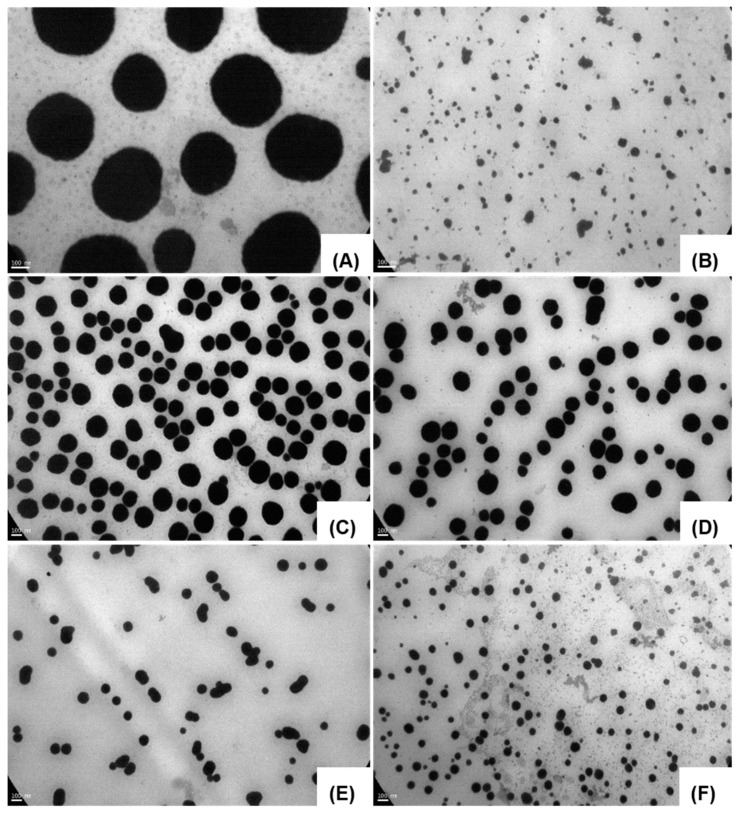
TEM images of pristine free-PMs (10% *w*/*v*): (**A**) F127, (**C**) T1307, and (**E**) T908 and co-associated PMs-probe (**B**) F127-probe, (**D**) T1307-probe, and (**F**) T908-probe. Scale bar: 100 nm. Notice the evident reduction in the particle sizes post co-association between the probe and PMs (**B**,**D**,**F**).

Images of the nanostructures in the presence or absence of the probe were acquired by TEM. Nanometric structures were observed in all cases (Figure 1 and Figure 2). It was possible to visualize PPL-probe and PMs-probe using a scale of 100 and 500 nm. Arrows in Figure 1D point out the probe on the surface of PPL-probe (similar to shallow ears on the surface). PPL-probe and PMs-probe showed clear differences respect to free-PPL and free-PMs, supporting that the co-association processes took place and the size values were well correlated with the DLS results (Table 1 and Table 2, Figure 1 and Figure 2).

We observed an evident reduction in particle sizes post co-association between probe and PMs (Figure 2B,D,F), which were well characteristic and consistent with DLS. Thereby, the greater stabilization of PMs based on PEO-PPO would occur in the presence of the probe, promoting the contraction of the micellar structures. These results were well distinguishable by DLS and TEM and were most noteworthy for T908-PMs (Table 2 and Figure 2 and Figure S3). This behavior was previously reported for other molecules [33,34]. PEO-PPO PMs are well known as smart materials, they self-assemble and micellized more adequately at body temperature and in presence of a cargo [31,32,33,34,35]. Furthermore, dehydration phenomenon over the hydration sphere of PMs at 37 °C shows a marked decrease in particle size by DLS and TEM (Table 2 and Figure 2) [33,34]. Finally, the stability of the nanosystem-probe followed by measuring the particle size and Z-potential was maintained for 24 h and stored in the refrigerator in the dark.

**Figure 3:**
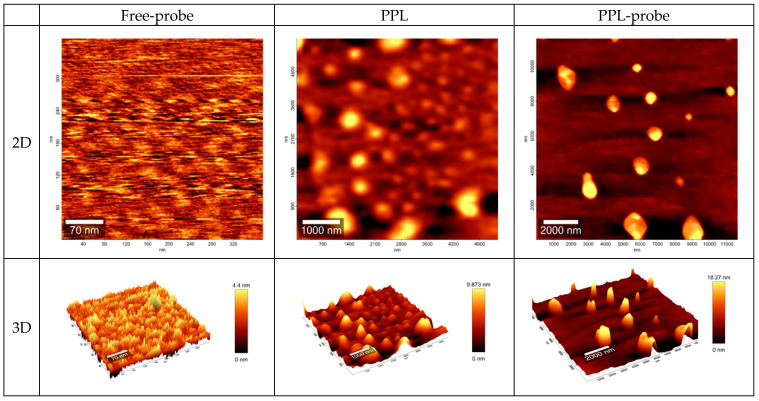
AFM images for topology in nm for two-dimensional (2D) and three-dimensional (3D) representation for free-probe, free-PPL, and PPL-probe. Note the well-marked spheres in PPL and PPL-probe.

**Figure 4:**
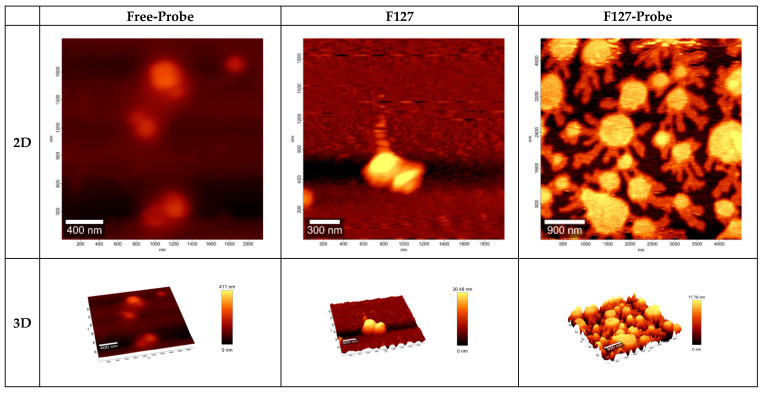

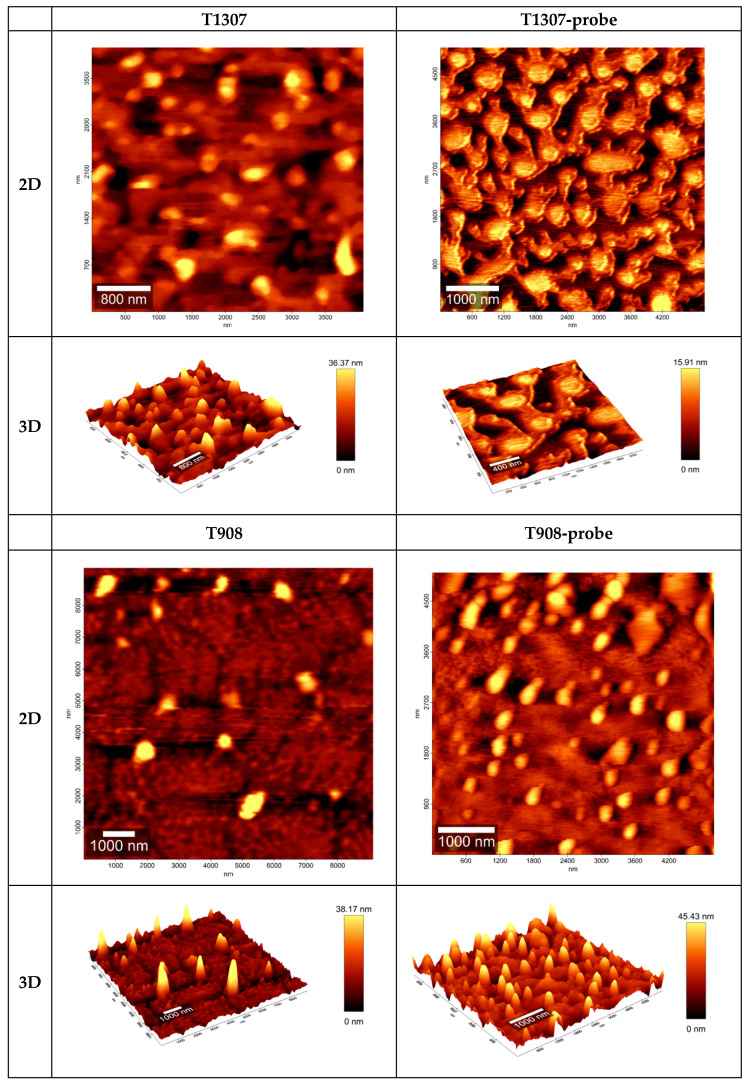
AFM images for topology in nm two-dimenstional (2D) and (three-dimensional (3D) representation for free-probe, F127, F127-probe, T1307, T1307-probe, T908, and T908-probe. Notice the off-surface arms on F127-probe PMs (2D, image on the upper right), which strongly suggest the probe on the surface.

In addition, the co-association between probe and PPL and PMs nanostructures was exhaustively studied by Atomic Force Microscopy (AFM) offering topographic, contrast, and morphologic information (see Figure 3 and Figure 4). 

Free-probe observed by AFM showed linear structures with variable high peaks until 4.3 nm. The free-PPL showed an oval structure with a 400 nm wide and 238 nm high (Figure 3). However, the co-association PPL-probe showed smaller structures with an approximately 25 nm size with irregular borders. It is possible to observe amplitude-contrast variations between PPL and PPL-probe, and we evidenced some structural or mechanical variations between regions, thus evidencing different material compositions while keeping the same topology (Figure 3). 

Additionally, and in concordance with the DLS and TEM results, PMs-probe presented smaller sizes tan free-PMs. The size for T908-probe was ≈212 nm lower than free-T908 PMs, which presented sizes between 319 and 616 nm by AFM (Figure 4). The observed height showed important differences, being more than twice the highest when comparing isolate probe with T908-probe (Figure 4). Even more, the off-surface arms on F127-probe PMs were evident, which strongly suggest the effectively probe on the surface (Figure 4, image on the upper-right). In the AFM, it is possible to observe two marked regions, one central region with a spherical core and accompanied by an irregular shell, suggesting a surface interaction between PMs and the probe. 

Finally, it is important to highlight that in microscopy techniques such as TEM and AFM, the nanostructures can be crushed or shrunk at the time of drying of the sample, and that is the reason they can have larger or smaller sizes compared to that studied by DLS.

### 3.4. In Vitro Probe Release from Nanosystems

**Figure 5:**
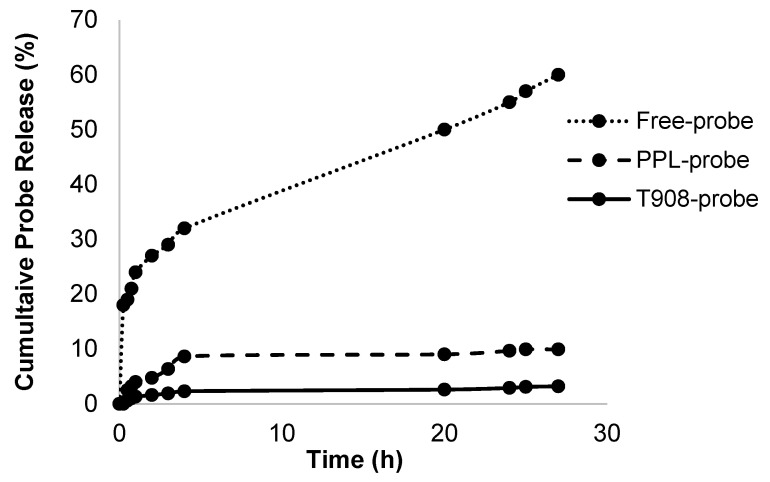
In vitro cumulative probe release percentage of free-probe, PPL-probe, and T908-probe, during 30 h. The concentration of probe was 50 µg/mL, and the sink conditions in the experiment were maintained in all cases.

In order to further study the stability of the co-association between the probe and the nanosystems, we studied the probe release profiles from PPL-probe and PMs-probe from *simil* biological media. The co-associated PPL-probe showed a slow release profile with approximately 10% of probe released after 24 h with a plateau (Figure 5). On the other hand, T908-probe PMs released only 3% during the first 30 h, with a plateau (Figure 5). A 60% of the free-probe was released after 24 h as control of the process (Figure 5). As we expected and as it was observed, the results for the PPL-probe and T908-probe did not show a burst effect or massive release of probe at short release times (Figure 5). The adjustment of kinetic release models revealed an adjustment to a first-order release and a prolonged release profile in both cases (Figure 5). This type of release profile was reported for other micellar nanostructures that we also studied before [34,49,50,51]. In addition, actually, approximately 75% of the modified-release profile in the pharmaceutical market coincides with first-order releases. 

However, it is important to remark that our main objective in this assay was to ensure the stability of the nanostructures–probe association and not study the release profile itself. The results we obtained here were totally compatible with in vivo imaging times.

### 3.5. Cellular Uptake of Probe Nanosystems

**Figure 6:**
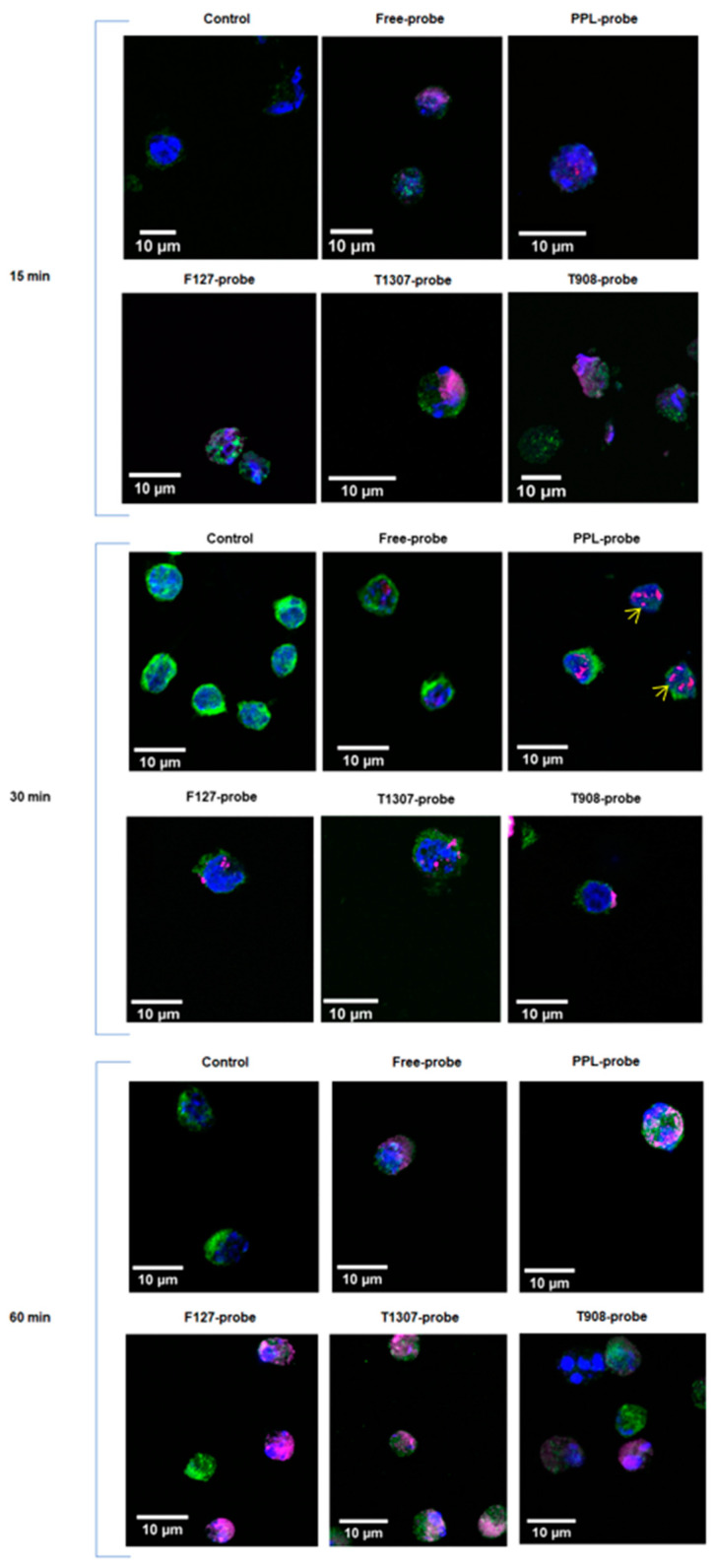
Confocal microscopy image of A20 cells, incubated for 15, 30, and 60 min with free-probe, PPL-probe, and PMs-probe: F127-probe, T1307-probe, and T908-probe. Yellow arrows show magenta nano-size structures corresponding with PPL-probe nanosystems. In the low panel, co-localization with the endosomal marker can be clearly observed.

Confocal microscopy images of A20 cells incubated with the co-associations are shown in Figure 6. The A20 cell line was incubated at 15, 30, and 60 min with the free-probe, PPL-probe, F127-probe, T1307-probe, and T908-probe. The nuclear and early endosomal markers were visualized using 405 nm (blue) and 488 nm (green) lasers, respectively, and the probe was visualized using a 640 nm (magenta) laser (Figure 6). After 30 min of incubation with the cells, the characteristic probe signals could be visualized for co-associated nanostructures, while for free-probe was not clearly observed. Surprisingly, magenta patterns with the size and morphology characteristics of the nanostructures were observed in the cells. In the PPL-probe images, several magenta spheres ≈500 nm were noticed (see arrows in Figure 6). In the PMs-probe images, smaller size spheres were observed and consistent with the reported values by TEM and AFM. After 60 min, it is possible to observe the co-localization between PPL-probe or PMs-probe, mainly in T1307-probe treatment by an endosomal marker. Thus, the endosomal route of cell uptake was confirmed. The increased uptake of nanosystem-probe was evident as was the most favored uptake of the nanosystems-probe in tracking. Additionally, at this time, the probe uptake in the cells is higher with PMs co-associations, which turned cells totally magenta. 

### 3.6. Biodistribution in A20 Tumor-Bearing Mice

On the one hand, ex vivo images were acquired 0.5, 2, and 24 h after injection of the probe or co-association PPL-probe. The ROI values of separated organs were analyzed, and results are described in the Supplementary Data (see Table S1). The tumor uptake was 723.0 ± 242.9 for the probe and 544.5 ± 139.1 for PPL-probe 2 h post-injection. The values for kidneys were 1041.8 ± 380.1 and 884.4 ± 283.1 for both the probe and PPL-probe, respectively. However, statistic non-significant differences were observed for both the probe and PPL-probe biodistribution (see Figure S4). In addition, tumor/non-target organs ratios did not show significant differences. 

**Figure 7:**
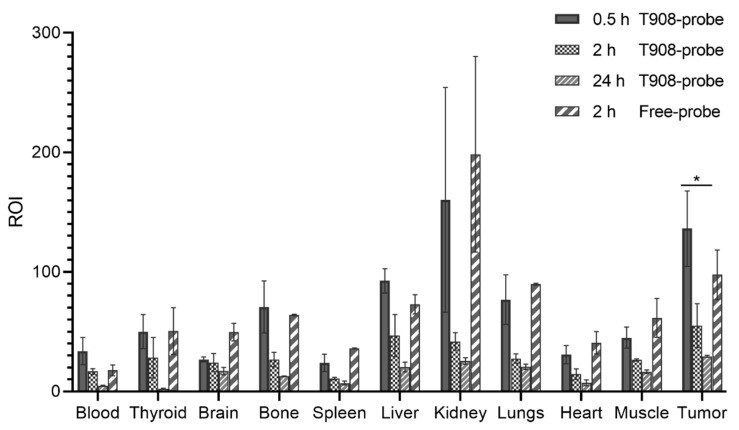
Biodistribution results for T908-probe at 0.5, 2, and 24 h post-injection in A20 tumor-bearing BALB/c mice. Control free-probe at 2 h post-injection. * *p* = 0.02 (Student’s *t*-test).

T908-probe nanosystem was also evaluated with ex vivo images acquired 0.5, 2, and 24 h post-injection. ROI values of separate organs are shown in Figure 7. The tumor uptake average values were 97.5 ± 20.7 and 55.0 ± 18.3 for 2 h post-injection with the probe and T908-probe, respectively (Figure 7). A significative difference was observed in kidney uptakes 2 h post-injection being the T908-probe ROI value in kidneys of 41.8 ± 7.3 and 198.5 ± 81.9 for the free-probe (Figure 7). In addition, a significant difference was observed for liver for the same time; the T908-probe ROI value was 46.7 ± 17.6, and it was 72.8 ± 8.0 for free-probe (Figure 7). These values and the tumor/blood ratio values for T908-probe are increasing over time (see Table S2).

### 3.7. Pharmacokinetic Studies in Mice

Based on our previous results, the pharmacokinetic study was performed with the T908-probe. The population pharmacokinetic analysis showed that the observations (ROI/µL) of the probe in blood throughout time were better described with a two-compartment disposition model, assuming first-order kinetics for both distribution and elimination from the central compartment. Mice body weight was included in each disposition parameter accounting for the size effect on probe distribution and elimination according to an allometric scaling model with a coefficient of 0.75 for elimination and distribution clearances, and 1 for the volume of distribution of the central and the peripheral compartment. Estimated parameters were elimination clearance (CL), distribution clearance (Q), volume of distribution of central compartment (V1), and volume of distribution of the peripheral compartment (V2). Inter-individual variability was included, assuming a log-normal distribution of individual parameters. The formulation effect in V2 was found to be significant (*p* < 0.01), with T908-probe having a 2.46-fold higher V2 relative to free-probe. Table 3 summarizes the estimated model parameters, while Figure S5 shows the VPC stratified by formulation (see Figures S5–S7). 

The increase in aptamer volume of distribution achieved by T908-probe led to significant differences in secondary exposure metrics such as elimination half-life (t_1/2_) and mean residence time (MRT) relative to free-probe. Typical values for these metrics were derived from the final pharmacokinetic model. T908-probe had a t_1/2_ of 5.6 h, versus 2.7 h for free-probe, and it had an MRT of 8.42 h versus 4.07 h for free-probe. 

## 4. Discussion

Aptamers have become an emerging class of biomolecules for target recognition. However, current gaps in diagnosis and treating cancer include loss of specificity, rapid drug clearance and biodegradation, and limited targeting. In the last few years, nanostructures have been used for biomedical applications [34,52,53]. Nanoparticles offer the opportunity to control the release such that a high percentage of the trapped drug is released after the particles have reached their target tissue. This property of controlled release from nanoparticles can improve the efficacy of the drugs while reducing off-target toxic effects [27,32,34,52]. Passive and active drug targeting with nanostructures can improve efficacy, reduce toxic side effects, and enhance the delivery of poorly soluble or sensitive therapeutic molecules [27,32,34,52,53]. Since the binding of aptamer to the corresponding target molecule depends on their correct folding [48], non-covalent surface interactions were explored to allow the easy target recognition in a nanosystem with multiple applications. Here, we describe a simple methodology of incubation to achieve the co-association with the pre-formed nanostructures and the Sgc8-c-Alexa647. Several methodologies were carried out to verify the interaction. 

A VEC protocol allows identifying and separating the fluorescent signal from the liposome fraction. More than 80% of the probe was observed in this fraction when PPL concentration was increased. Those results, with AFM and confocal microscopy data, confirm the co-association between the PPLs and the probe. Differences in morphology and material, as well as the size of the nanosystems observed in these experiments were correlated. The in vitro release experiment showed a slow release profile of the probe from PPL-probe with first-order kinetic adjustment, which encouraged us to perform in vivo experiments. The experiments of PPL-probe in tumor-bearing mice did not show significant differences in the biodistribution profile for the evaluated times. According to these results, the co-association did not promote changes in the biodistribution profile. In vivo experiments are limited by the sensitivity of the fluorescence images, and we are encouraged to include our radioactive probe to have low background, additional time points, and the possibility to follow in vivo images during the time. 

We also evaluated PMs–probe co-associations. D*_h_* and Z-potential showed significant differences between free-probe and the nanosystems-probe, and TEM and AFM images confirmed the contraction effect reported before. The AFM–amplitude values also showed differences in the surface materials of the free PMs and PMs-probe. In confocal microscopies images, we could observe small fluorescent nanosized structures over the cells, at 30 min after incubation, for all PMs assessed (Figure 6). Due to the PEO/PPO composition, the homogeneous size distribution, and smaller size, we selected the T908-probe co-association to perform the release experiment, which indicated a first-order kinetics. However, it is important to highlight that our main objective in the vitro release assay was to ensure the stability of the nanostructure–probe associations. The results obtained were compatible with in vivo imaging times.

Surprisingly, the in vivo biodistribution of T908-probe showed significant differences in liver and kidney uptakes compared to the free-probe, which was in agreement with our previous report. Those differences could indicate more circulating time of the co-associated T908-probe [32,33,34,35]. It can enhance active delivery due to the interaction of the probe with the specific receptor and, added to a passive delivery due to the nanometric sizes of the micellar nanosystems, themselves.

Thus, PMs based on T908-probe showed greater permanence in circulation in tumor-bearing mice compared to free-probe, and it was consistent with the found pharmacokinetics parameters. In the same direction, the pharmacokinetic analysis of aptamer fluorescence in blood showed a significant effect of T908-probe increasing the aptamer distribution to peripheral spaces. Although the co-associated probe does not change the aptamer elimination clearance from blood, the estimated 2.46-fold higher volume of distribution of the peripheral compartment relative to free-probe resulted in a significantly higher mean residence time (2.07-fold). Therefore, the increased permanence of the probe in the body when administered as a co-association T908-probe is based on a boosted distribution into extravascular sites. Further studies can contribute to understand the nature of the nanostructure and probe interaction.

## 5. Conclusions

Here, we demonstrate that it is possible to co-associate aptamer probes with different nanostructures. Particularly, the co-association T908-probe showed a different and desirable biodistribution and pharmacokinetic profile, which could contribute to the permanence in the organism and the passive targeting delivery into a tumoral microenvironment.

## Figures and Tables

**Table 1:** Hydrodynamic diameter (D*_h_*), size distribution (PDI), and Z-potential of free-probe (50 μg/mL), free-PPL and PPL-probe post-incubation (PPL and PPL-probe), and post-incubation and purification (PPL-c and PPL-probe-c) by vesicular exclusion column (VEC) as measured by DLS in Milli-Q water at 25 and 37 °C. The results were expressed as the mean ± S.D. of at least six runs (n = 3). The software used for the analysis of the data was v7.12 software (Malvern Instruments).

Samples	Temp. (°C)	Z-Average (nm)	Size Distribution by Intensity (%)			
			D*_h_* (nm) (±S.D.)	% Intensity (±S.D.)	PDI	Z-Potential (mV) (±S.D.)
Free-probe	25	12.9 (2.2)	26.6 (3.4)	100.0 (0.0)	0.417 (0.114)	−12.8 (2.6)
PPL		189.8 (6.3)	249.3 (20.2)	100.0 (0.0)	0.403 (0.031)	−35.2 (0.6)
PPL-probe		236.4 (8.2)	263.9 (8.4)	100.0 (0.0)	0.401 (0.072)	−31.3 (0.5)
PPL-c		171.6 (3.2)	213.0 (15.7)	100.0 (0.0)	0.379 (0.017)	−64.9 (0.8)
PPL-probe-c		186.6 (4.8)	241.4 (19.1)	100.0 (0.0)	0.363 (0.033)	−64.9 (0.8)
Free-probe	37	11.8 (0.2)	42.1 (11.8)	100.0 (0.0)	0.405 (0.016)	−8.0 (0.7)
PPL		202.1 (11.5)	256.6 (17.6)	100.0 (0.0)	0.404 (0.056)	−33.5 (0.7)
PPL-probe		176.2 (17.0)	192.0 (18.9)	100.0 (0.0)	0.151 (0.056)	−28.9 (0.7)
PPL-c		184.2 (7.7)	221.1 (14.6)	100.0 (0.0)	0.420 (0.045)	−60.7 (1.0)
PPL-probe-c		203.9 (6.0)	226.1 (19.3)	100.0 (0.0)	0.391 (0.043)	−64.0 (2.9)

**Table 2:** Hydrodynamic diameter (D*_h_*), size distribution (PDI), and Z-potential of pristine PMs: F127, T1307, and T908 and PMs-probe: F127-probe, T1307-probe, and T908-probe, as measured by DLS at 25 and 37 °C. The results were expressed as the mean ± S.D. of at least six runs (n = 3). The software used for the analysis of the data was v7.12 software (Malvern Instruments).

PMs (10% *w*/*v*)	Temp. (°C)	Peak 1		Peak 2		PDI (±S.D.)	Z-Potential (mV) (±S.D.)
		D*_h_* (nm) (±S.D.)	% Intensity (±S.D.)	D*_h_* (nm) (±S.D.)	% Intensity (±S.D.)		
F127	25	** 44.1 (3.1)	86.4 (1.3)	* 5.3 (0.2)	13.3 (1.3)	0.473 (0.013)	−3.7 (0.3)
F127–probe		** 31.8 (7.3)	100.0 (0.0)	--	--	0.447 (0.058)	−3.0 (0.8)
F127	37	** 21.5 (0.3)	96.2 (2.7)	--	--	0.271 (0.034)	−3.0 (0.7)
F127–probe		** 30.4 (0.9)	100.0 (0.0)	--	--	0.487 (0.068)	−2.2 (0.5)
T1307	25	** 64.4 (1.8)	53.7 (1.5)	* 6.9 (0.5)	46.3 (1.5)	0.551 (0.081)	−5.0 (0.3)
T1307–probe		** 24.0 (3.2)	81.2 (8.8)	*** 170.5 (58.0)	18.8 (8.8)	0.459 (0.080)	−4.4 (0.6)
T1307	37	** 18.9 (0.8)	100.0 (0.0)	--	--	0.331 (0.067)	−4.6 (0.5)
T1307–probe		** 25.9 (5.1)	100.0 (0.0)	--	--	0.450 (0.024)	−2.6 (0.4)
T908	25	*** 174.3 (23.1)	47.7 (0.7)	* 6.4 (0.8)	52.3 (0.7)	0.502 (0.076)	−7.9 (0.6)
T908–probe		** 10.7 (0.1)	93.6 (1.3)	*** 166.4 (9.1)	6.4 (1.3)	0.447 (0.018)	−3.4 (0.5)
T908	37	** 89.6 (13.3)	47.9 (2.8)	* 8.6 (0.1)	52.1 (2.8)	0.305 (0.055)	−6.7 (0.6)
T908–probe		** 19.8 (1.9)	100.0 (0.0)	--	--	0.264 (0.092)	−2.4 (0.3)

* Unimers, ** PMs and *** Micellar aggregates.

**Table 3:** Typical parameter values are reported for an animal with 25 g body weight. IIV stands for inter-individual variability, RUV stands for residual unexplained variability (described with an additive error model), and RSE (%) stand for the relative standard error quantifying the uncertainty in parameter estimation. Pharmacokinetic parameters: elimination clearance (CL), distribution clearance (Q), volume of distribution of the central compartment (V1), and volume of distribution of the peripheral compartment (V2).

Parameter	Value	RSE (%)
CL (mL/min)	0.158	27.9
V1 (mL)	10.4	13.9
Q (mL/min)	0.585	11.0
V2 free-probe (mL)	22.3	20.2
V2 T908-probe (mL)	54.8	35.3
IIV CL (%)	83.8	26.7
IIV V1 (%)	45.1	23.3
IIV Q (%)	30.7	28.1
IIV V2 (%)	32.8	46.8
RUV (ROI/µL)	3.9	12.8

## Data Availability

The data presented in this study are available in this article.

## Supplementary Materials

The following supporting information can be downloaded at: https://www.mdpi.com/article/10.3390/ph15010015/s1, Figure S1. (A) Representative chemical structure of Sgc8c-Alexa647 probe. Red indicates the principal domain from the fluorophore portion Alexa647 with hydrophobic characteristics, and light blue indicates the hydrophilic domain from the oligonucleotide structure. In this work, we propose exhaustively investigating its co-association with two structurally different types of nanostructures (PPL and PMs) at different hydrophilic and hydrophobic key points of these nanosystems. (B) Schematic figure of the proposed hypothesis regarding the possible interactions of the aptamer with each studied nanosystem (PPL and PMs). Figure S2. Chemical formula of pristine copolymers used in this work: (A) lineal poloxamer F127 and (B) branched poloxamine T1307 and T908. Table S1. Biodistribution data from ex vivo images of PPL-probe and free-probe (control). Images were acquired 0.5, 2, and 24 h post injection of PPL-probe and 2 h after free-probe injection in A20 tumor-bearing mice (n = 5). Values are in ROI. Figure S3. Average size distributions by intensity (%) (left) and average graphs of correlation coefficients as a function of time (μs) for free-PMs (F127, T1307, and T908) and PMs-probe (F127-probe, T1307-probe, and T908-probe) (right) at 37 °C, by DLS. Note the correlation coefficient data for PMs-probe in all cases with characteristic noise due to the presence of the fluorescent probe. Each curve was plotted as the average of six determinations using the v7.12 software (Malvern Instruments). Figure S4. Biodistribution results for PPL-probe at 0.5, 2, and 24 h post-injection in A20 tumor-bearing BALB/c mice. Control free-probe at 2 h post-injection. Table S2. Biodistribution data from ex vivo images of T908-probe and free-probe (control). Images were acquired 0.5, 2, and 24 h post injection of T908-probe and 2 h after free-probe injection in A20 tumor-bearing mice (n = 5). Values are in ROI. Figure S5. Individual fits for the final model in logarithmic scale. Blue dots correspond to the observations of aptamer in blood (ROI/µL), while the purple line reflects the model-based pharmacokinetic curve. Figure S6. Visual predictive check (VPC) for the final model. Observations are shown as dots. The blue line stands for the median of the observations. The black dashed line represents the model-based predicted median, and the blue area is the corresponding 95% prediction interval. Overall, the VPC shows a good fit of the data obtained after the administration of both formulations. Figure S7. Observations vs. individual predictions. Blue dots indicate the measured aptamer fluorescence in blood. The identity line (slope = 1, intercept = 0) is shown as a black line.
