# Supplementary material for: T908 Polymeric Micelles Improved the Uptake of Sgc8-c Aptamer Probe in Tumor-Bearing Mice: A Co-Association Study between the Probe and Preformed Nanostructures
Source: Pharmaceuticals (Basel). 2021 Dec 23;15(1):15. doi: 10.3390/ph15010015 (PMC8780797; doi:10.3390/ph15010015)
Supplement: Supplementary file 1 [file pharmaceuticals-15-00015-s001.zip › pharmaceuticals-1477720-supplementary.pdf]

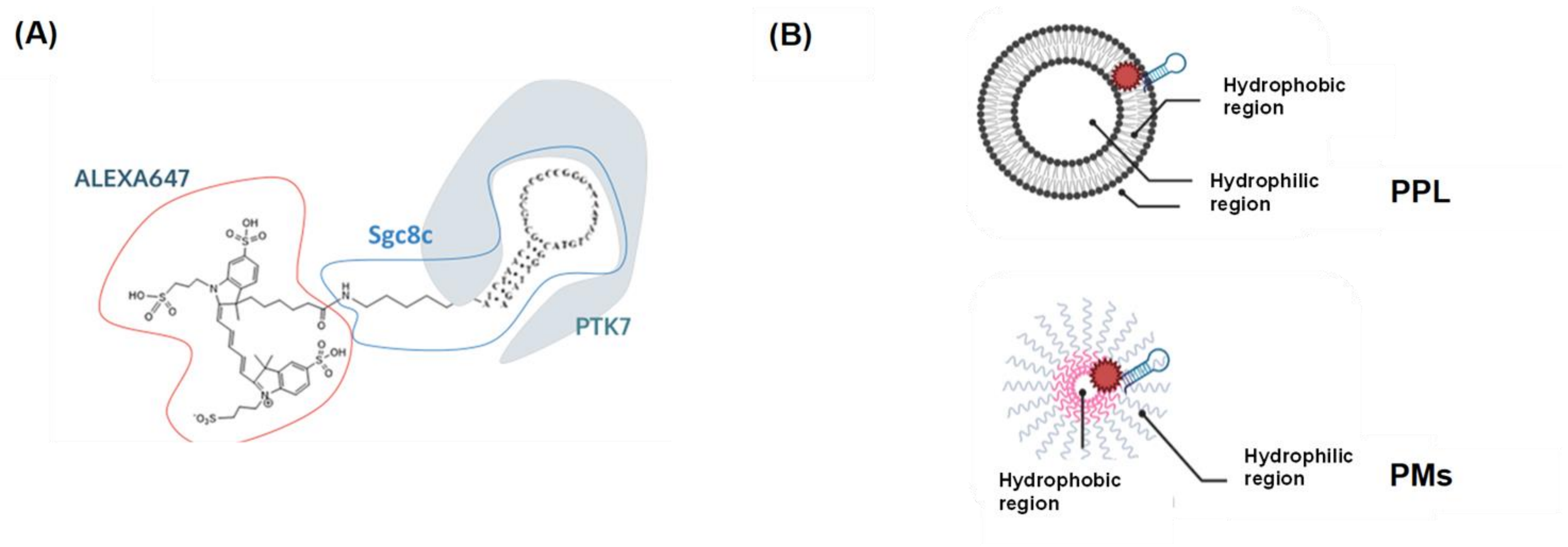

Figure S1. (A) Representative chemical structure of Sgc8c-Alexa647 probe. In red, it is indicated the principal domain from the fluorophore-portion Alexa647 with hydrophobic characteristics and in light blue, it is indicated the hydrophilic domain from the oligonucleotide structure. In this work we propose to exhaustively investigate its co-association with two structurally different type of nanostructures (PPL and PMs) at different hydrophilic and hydrophobic key-points of these nanosystems. (B) Schematic figure of the proposed hypothesis regarding the possible interactions of the aptamer with each studied nanosystem (PPL and PMs).

(A)

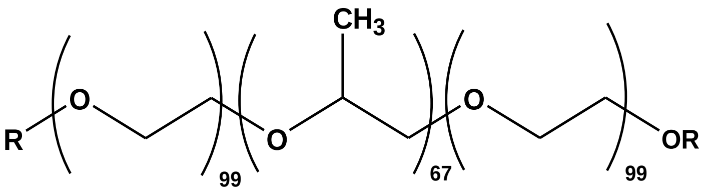

F127-Glu

R = H

(B)

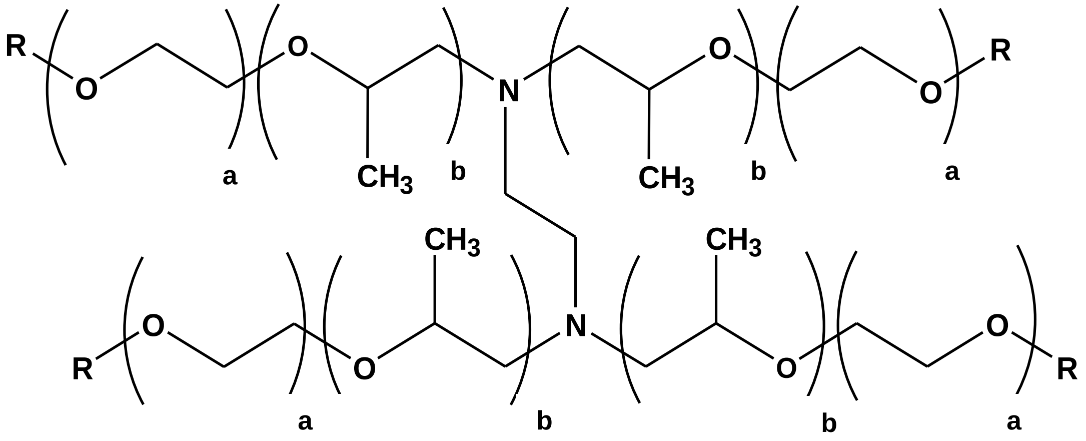

**T1307**	**a = 72**	**b = 23**
**T908**	**a = 114**	**b = 21**
**R = H**		

Figure S2. Chemical formula of pristine copolymers used in this work: **(A)** lineal poloxamer F127 and **(B)** branched poloxamine T1307 and T908.

Table S1. Biodistribution data from ex vivo images of PPL-probe and free-probe (control). Images were acquired 0.5, 2, 24 h post injection of PPL-probe and 2 h after free-probe injection in A20 tumor bearing mice (n=5). Values are in ROI.

Organ/Tissue	0.5 h			2 h			24 h			2 h control		
Blood	284.7	±	134.6	207.6	±	74.1	281.7	±	11.8	210.4	±	3.7
Thyroid	256.9	±	109.2	232.3	±	91.4	188.0	±	2.1	210.4	±	17.3
Brain	297.2	±	63.4	325.5	±	40.8	237.9	±	11.2	297.5	±	13.3
Bone	611.4	±	214.3	427.9	±	84.1	209.4	±	12.4	402.0	±	58.7
Spleen	317.3	±	92.3	289.4	±	6.8	238.1	±	22.2	377.1	±	74.5
Liver	743.8	±	60.0	642.0	±	18.9	397.9	±	48.1	653.9	±	31.0
Kidneys	935.3	±	192.2	884.4	±	283.1	719.2	±	219.6	1041.8	±	380.1
Lungs	698.7	±	359.0	388.8	±	126.1	212.6	±	22.3	495.8	±	139.8
Heart	330.9	±	173.2	253.7	±	77.8	166.9	±	25.6	255.9	±	27.4
Muscle	540.5	±	242.9	311.6	±	100.4	147.2	±	20.4	399.6	±	68.8
Tumor	1042.8	±	65.9	544.5	±	139.1	338.1	±	30.6	723.0	±	242.9
**target/non-target ratio**												
Tumor/blood	4.18	±	2.21	2.75	±	0.88	1.00	±	0.29	3.43	±	1.09
Tumor/Kidneys	1.15	±	0.31	0.63	±	0.06	0.51	±	0.20	0.70	±	0.02
Tumor/lungs	1.75	±	0.99	1.18	±	0.31	1.61	±	0.32	1.45	±	0.08
Tumor/liver	1.41	±	0.20	0.84	±	0.20	0.85	±	0.08	1.12	±	0.42
Tumor/muscle	2.18	±	1.10	1.78	±	0.17	2.35	±	0.54	1.89	±	0.93

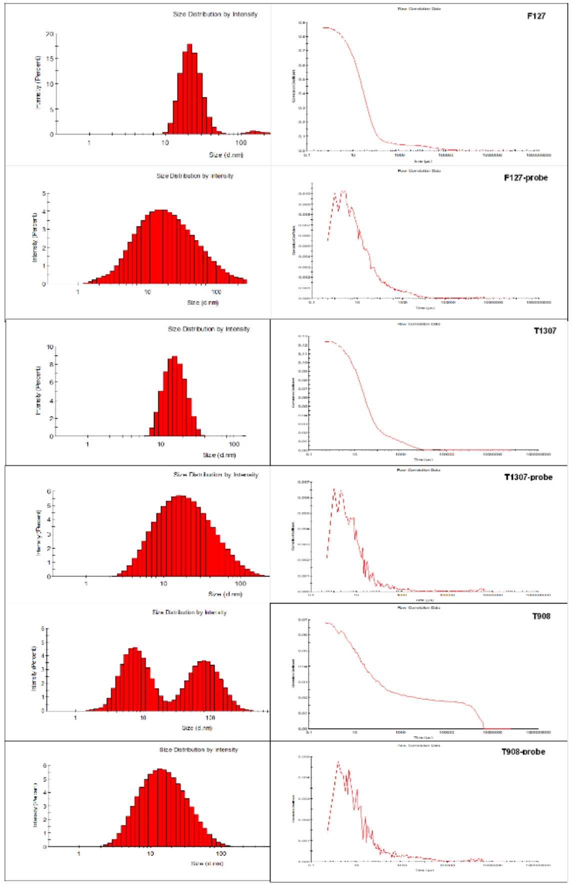

Figure S3. Average size distributions by Intensity (%) (left) and average graphs of correlation coefficients as a function of time (μs) for free-PMs (F127, T1307 and T908) and PMs-probe (F127-probe, T1307-probe and T908-probe) (right) at 37ºC, by DLS. Note the correlation coefficient data for PMs-probe in all cases with characteristic noise due to the presence of the fluorescent probe. Each curve was plotted as the average of six determinations using the v7.12 software (Malvern Instruments).

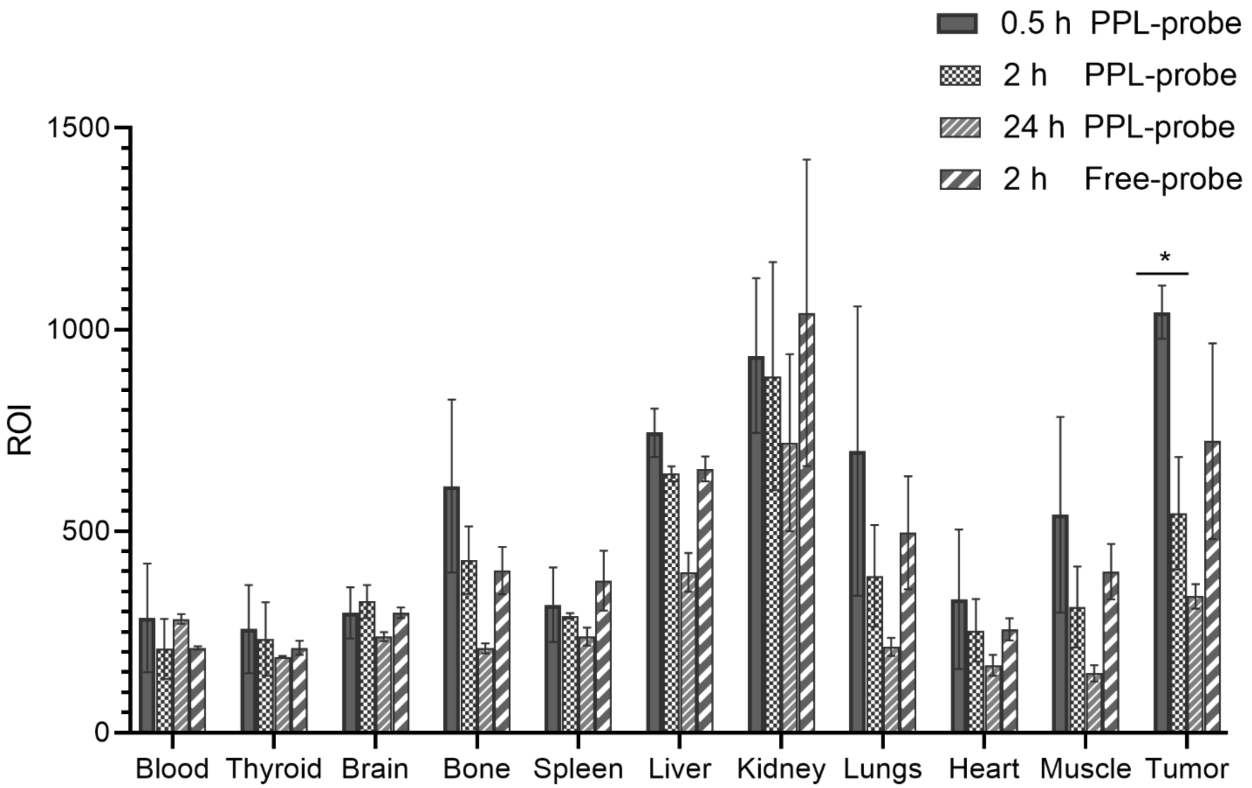

Figure S4. Biodistribution results for PPL-probe at 0.5, 2 and 24 h post-injection in A20 tumor bearing BALB/c mice. Control free-probe at 2 h post-injection.

Table S2. Biodistribution data from ex vivo images of T908-probe and free-probe (control). Images were acquired 0.5, 2, 24 h post injection of T908-probe and 2 h after free-probe injection in A20 tumor bearing mice (n=5). Values are in ROI.

Organ/Tissue	0.5 h			2 h			24 h			2 h control		
Blood	33.69	±	11.34	16.75	±	2.29	4.76	±	0.44	17.66	±	4.50
Thyroid	50.10	±	14.29	28.16	±	17.00	2.30	±	0.31	50.51	±	19.51
Brain	26.82	±	2.10	24.16	±	7.59	17.04	±	3.11	49.51	±	7.34
Bone	70.71	±	21.74	26.67	±	6.00	12.63	±	0.30	63.97	±	0.52
Spleen	23.83	±	7.27	10.79	±	1.00	7.07	±	1.59	35.95	±	0.38
Liver	92.49	±	10.21	46.70	±	17.62	20.44	±	4.14	72.82	±	8.00
Kidneys	160.31	±	93.98	41.78	±	7.32	25.40	±	2.80	198.47	±	81.93
Lungs	76.74	±	20.90	27.48	±	3.86	20.64	±	2.26	89.69	±	0.66
Heart	30.79	±	7.61	14.52	±	4.39	7.22	±	2.39	40.72	±	9.35
Muscle	44.96	±	8.84	26.33	±	0.96	16.51	±	1.50	61.53	±	16.35
Tumor	136.06	±	31.49	55.03	±	18.30	29.31	±	0.79	97.54	±	20.70
**target/non-target ratio**												
Tumor/blood	4.49	±	1.41	3.99	±	1.25	6.09	±	0.55	5.17	±	1.68
Tumor/Kidneys	0.89	±	0.35	1.49	±	0.67	1.17	±	0.17	0.56	±	0.34
Tumor/lungs	1.83	±	0.48	1.91	±	1.00	1.43	±	0.13	1.09	±	0.22
Tumor/liver	1.47	±	0.26	0.83	±	0.15	1.47	±	0.30	1.36	±	0.43
Tumor/muscle	1.96	±	0.74	2.36	±	0.67	1.78	±	0.17	1.69	±	0.79

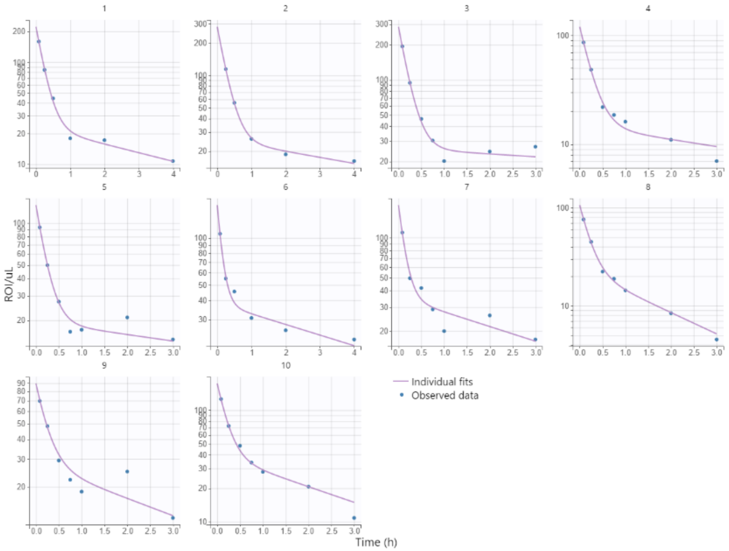

Figure S5. Individual fits for the final model in logarithmic scale. Blue dots correspond to the observations of aptamer in blood (ROI/µL) while the purple line reflects the model-based pharmacokinetic curve.

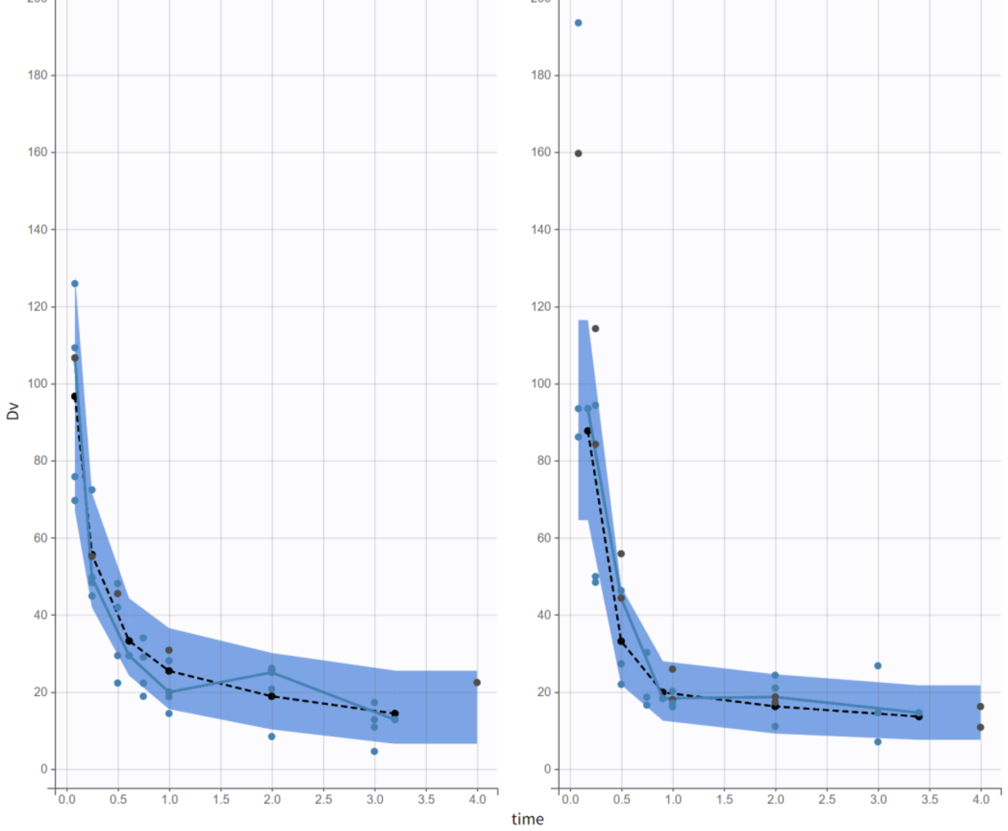

Figure S6. Visual predictive check (VPC) for the final model. Observations are shown as dots. The blue line stands for the median of the observations. Black dashed line represents the model-based predicted median, and the blue area is the corresponding 95% prediction interval. Overall, the VPC shows a good fit of the data obtained after administration of both formulations.

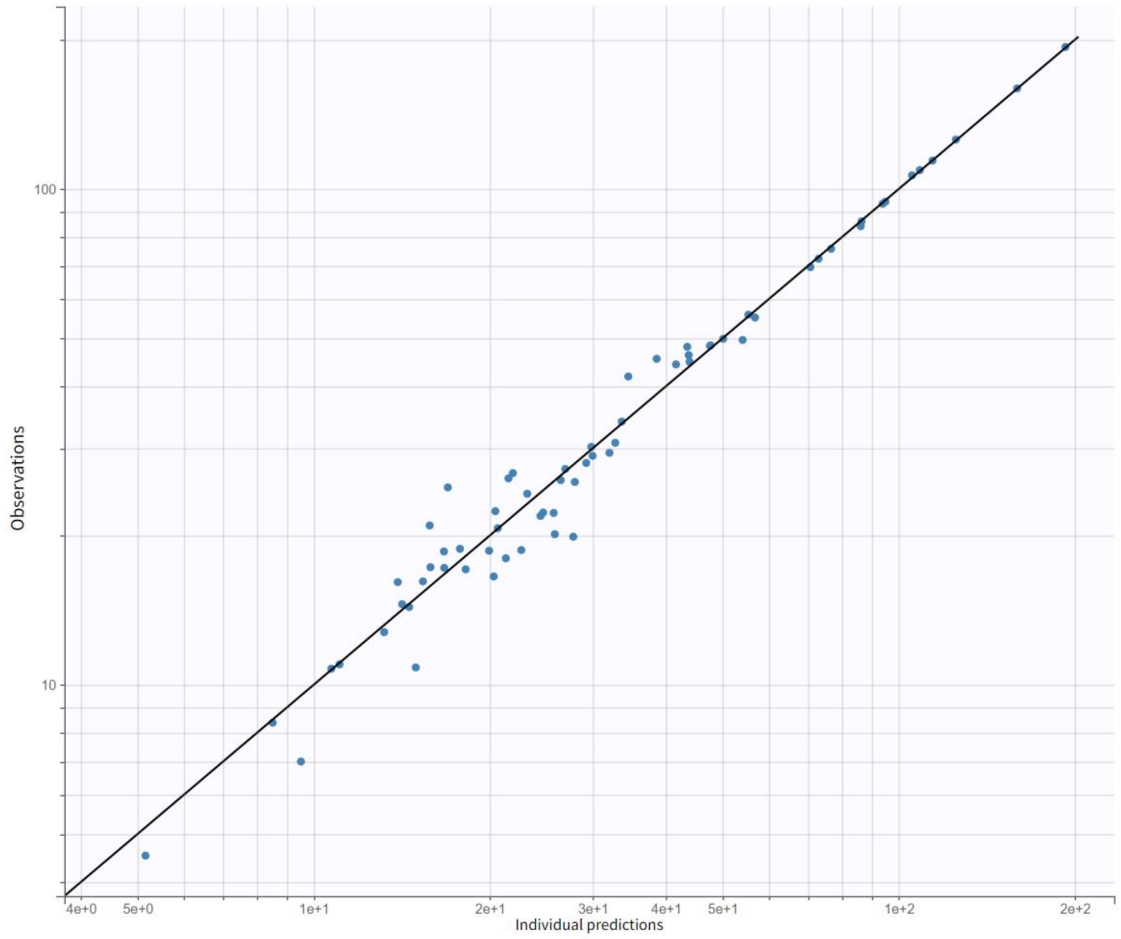

Figure S7. Observations vs Individual predictions. Blue dots are measured aptamer fluorescence in blood. The identity line (slope=1, intercept = 0) is shown as a black line.
